# Moving Forward: Understanding Correlates of Physical Activity and Sedentary Behaviour during COVID-19 in Children and Adolescents—An Integrative Review and Socioecological Approach

**DOI:** 10.3390/ijerph19031044

**Published:** 2022-01-18

**Authors:** Rachel L. Knight, Melitta A. McNarry, Adam W. Runacres, James Shelley, Liba Sheeran, Kelly A. Mackintosh

**Affiliations:** 1 Applied Sports, Technology, Exercise and Medicine (A-STEM) Research Centre, Swansea University, Swansea SA1 8EN, UK; 974302@swansea.ac.uk (R.L.K.); M.Mcnarry@Swansea.ac.uk (M.A.M.); 918800@swansea.ac.uk (A.W.R.); James.Shelley@swansea.ac.uk (J.S.); 2 School of Healthcare Sciences, College of Biomedical and Life Sciences, Cardiff University, Cardiff CF14 4EP, UK; sheeranl@cardiff.ac.uk; 3 Biomechanics and Bioengineering Research Centre Versus Arthritis, Cardiff University, Cardiff CF10 3AX, UK

**Keywords:** physical inactivity, youth, coronavirus, young people, sedentary time, movement behaviours, SARS-CoV-2, determinants, COM-B model, behaviour change

## Abstract

Novel coronavirus disease 2019 (COVID-19) pandemic restrictions have negatively impacted physical activity (PA) and sedentary time/behaviour. This integrative review systematically explored the socioecological factors that impacted and influenced these movement behaviours in children and adolescents during the pandemic. Five electronic databases were systematically searched in January 2021, with data extracted from 16 articles (*n* = 18,352; 5–17 years; 12 countries). Risk-of-bias was assessed using the Mixed Methods Assessment Tool (MMAT), with correlates identified, coded, and themed via thematic analysis. A socioecological model of during-pandemic PA and sedentary time/behaviour was conceptualised and mapped to the Capability, Opportunity, Motivation, and Behaviour (COM-B) model of behaviour-change mechanisms, illustrating influences over five levels: Individual (biological)—age and sex; Individual (psychological)—mental health, and cognition, motivation, and behaviour; Social—family factors, and structured support; Environmental—area of residence and resources; and Policy—COVID-19-related rules. For sedentary time/behaviour, individual-(age and sex), social-(family factors) and policy-(COVID-19-related rules) level factors may be important correlates. There were no age or sex associations with PA levels, though there was some indication that sedentary time/behaviour increased with age. Interventions seeking to enhance young people’s movement behaviours during periods of enforced restrictions should focus on enhancing opportunities on a social and environmental level.

## 1. Introduction

The challenges that surround effectively addressing physical inactivity and prolonged sedentary time/behaviour in children and adolescents are well documented [1]. It is recommended that children and adolescents engage in an average of 60 min of moderate-to-vigorous physical activity (MVPA) daily, including vigorous-intensity activities, as well as activities that strengthen muscle and bone at least three times per week [2]. However, more than 80% of adolescents globally do not meet these recommendations [3] and this lack of physical activity (PA) is typically accompanied by high volumes sedentary time/behaviour. Indeed, a recent international study suggests that children and adolescents spend between 246 and 387 min⋅day^−1^ sedentary, respectively [4]. These estimates are currently particularly concerning given the potential role of PA in reducing the risk of community-acquired infections, including the novel coronavirus disease 2019 (COVID-19) [5].

COVID-19, caused by infection with the severe acute respiratory syndrome coronavirus 2 (SARS-CoV-2), was first reported in December 2019. On declaring it a global pandemic on 11 March 2020, the World Health Organization (WHO) directed countries to implement strategies to identify, manage, and curtail the spread and impact of the virus [6]. Whilst responses varied globally, national responses included the closure of education, sports and recreational facilities, social distancing, restrictions on travel, and the requirement to self-isolate and quarantine. Our knowledge and understanding of the effects of these restrictions on the health and well-being of children and adolescents is, given the rarity of such events, understandably limited. However, the necessary global restrictions imposed to control the spread of COVID-19 are likely to have exacerbated the issue of physical inactivity and highly sedentary behaviours [7].

The evidence surrounding the impact of sedentary behaviour, defined as any energy expenditure ≤ 1.5 metabolic equivalents (METs) whilst awake and in a seated or reclining posture [8], on children and adolescents’ health, is not as robust as in adult populations. Nonetheless, given it is an independent risk factor for mortality among adults meeting the PA guidelines [9], it may be imperative to establish ways to limit the embodiment and internalization of such negative behaviours in children and adolescents during maturation processes. The adoption of more positive lifestyle habits that prevent and limit the impact of poor health may indeed facilitate the success of future public health initiatives and pandemic curtailment strategies.

On an individual and population level, PA is a complex and multi-faceted behaviour. Multiple interacting levels of variables, as outlined within the socioecological model of Sallis et al. [10], have the potential to direct, influence and facilitate “*active*” behaviours. Understanding which conditions need to be met or challenged for targeted behaviour change, as per the Capability, Opportunity, Motivation, and Behaviour (COM-B) model of behaviour change [11], and on what level [12], will not only enable us to build an understanding of the wider impact on children and young people of the COVID-19 pandemic, but may also inform subsequent interventions and policy strategies. Indeed, according to the COM-B model, the behaviour change system that occupies the central position of the Behaviour Change Wheel (BCW), one or more of three potential mechanisms (capability, opportunity, motivation) need to be targeted for effective change to occur [11]. The socioecological approach determines that these changes need to occur on an individual, social, environmental and/or policy level [10].

Through the interrogation of available literature on the correlates of PA and sedentary time/behaviour during the COVID-19 pandemic, this integrative review aimed to: (i) establish the impact of the COVID-19 pandemic on the socioecological correlates of PA and sedentary time/behaviour in children and adolescents and; (ii) map the identified correlates against the COM-B model to highlight which mechanisms of behaviour change could be the most pertinent to challenge physical inactivity in children and adolescents as we transition to a new normal. The derived model will enable policy makers globally to make recommendations and feed into effective responses and public health interventions.

## 2. Materials and Methods

### 2.1. Literature Review Method

In line with published guidance [13], an integrative review of both quantitative and qualitative literature relating to COVID-19, PA, sedentary time and behaviour was conducted. On 16 January 2021, electronic databases (EBSCOhost Medline, CINAHL plus, EBSCOhost SPORTDiscus, SCOPUS, Web of Science) were used to search key terms. Boolean and MeSH terms, developed following librarian guidance, were used to search for the following terms and variations of each term; *“Physical activity”,* *“Exercise”, “Sport”, “Recreation”, “Active travel”, “Physical performance”, “Physical function”, “Sedentary time” “Sedentary behaviour”, “Sedentary lifestyle”, “Physical inactivity”, “Prolonged sitting”,* and *“Coronavirus”, “COVID-19”, “SARSCov2”, “n-CoV”,* and *“Novel coronavirus”.* For a list of the full search terms, see online Supplementary File S1. To ensure that all relevant articles were captured, the population-level criteria were not applied until full-text screening. Given the number of extracted full texts, the population was split into adults and older adults (≥18 years) [14], which is reported separately and provides further methodological details, and children and adolescents aged 5–17 years. However, briefly, this integrative review included studies published in English which assessed correlates of PA and sedentary time/behaviour in children and adolescents (aged 5–17 years) during the COVID-19 pandemic. All generated citations and abstracts were independently reviewed using Rayyan (QCRI, Qatar) by two authors (RLK and AWR) to select eligible studies, followed by subsequent full-text screening, against the pre-defined inclusion/exclusion criteria (Table 1). No disagreements regarding eligibility arose. 

A standardised database, based on the Cochrane Collaboration guidance [15], was piloted, and used to extract data from the included studies for evidence synthesis. Extracted information included: authors; year of publication; study design, setting, population including sample size; participant demographics and characteristics; study methodology and recruitment; inclusion/exclusion criteria; context of the pandemic to include stage, mitigation, and confinement strategies in place at the time of the study, and correlates of PA and sedentary time/behaviour in relation to the COVID-19 pandemic. A second unblinded reviewer (LS) independently reviewed 25% of the extracted data. Supplementary data was consulted where available and necessary. 

### 2.2. Quality Assessment

Given that the critical appraisal of the literature is now deemed crucial for an integrative review [13], the Mixed Methods Assessment Tool (MMAT) [16], suitable for assessing different study designs, (quantitative – descriptive, randomised, and non-randomised trials; qualitative; mixed methods), was used to rate the appropriate research domain criteria as “Yes”, “No”, or “Unclear”. One author (RLK) independently appraised the quality of each included study, with a second author (AWR) randomly checking 25% of the ratings to ensure consistency. Each study was attributed an overall quality score, presented using asterisks (*) as a descriptor, from 1* (where 20% of the quality criteria were met) to 5* (where 100% of the quality criteria were met) [17]; no studies were excluded due to low quality.

### 2.3. Data Analysis and Model Development

Thematic analysis, using the six-stage process of Braun and Clarke [18], was undertaken by the first author (RLK) who identified correlates of PA and sedentary time/behaviour during the COVID-19 pandemic from the extracted data. Codes were allowed to emerge inductively from the semantic meaning of the data under the deductive headings *Individual*, *Social*, *Environmental*, and *Policy*, as identified from the socioecological model of Sallis et al. [10]. A “critical friend” (LS) independently challenged the generated codes and categorised sub-themes, named, and defined, to accurately represent the data, checking back against the original data extracts. Refinements were made where necessary to ensure congruity. 

Using the generated sub-themes, the first author (RLK) subsequently completed a two-step process, involving the conceptualisation of a COVID-19 context-specific socioecological model consistent with parameters of Sallis et al. [10], and then mapping the model against the behaviour mechanisms of the COM-B model [11]. The “critical friend” then blindly cross-matched 10% of the studies against the generated model to ensure consistency in approach, that the data had been mapped appropriately, and to enhance the transparency, credibility, quality control, and rigour of the processes undertaken [19]. Four discrepancies were discussed and reviewed in reverse, from the model to the original studies, until a consensus was reached.

## 3. Results

**Figure 1:**
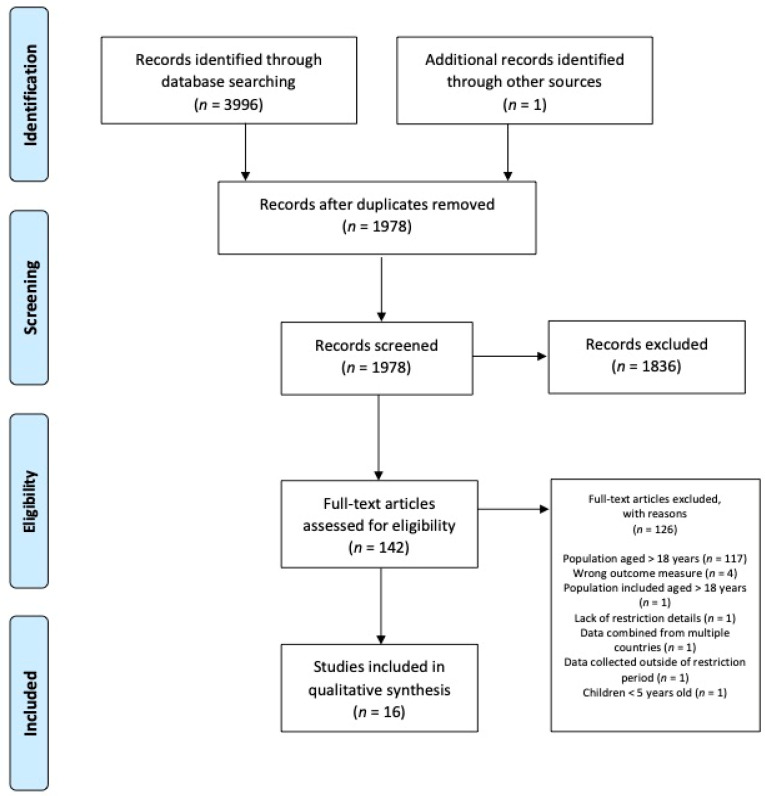
Schematic flow diagram of the integrative review process.

In total, 3997 articles were identified from database (*n* = 3996) and secondary population-specific searches (*n* = 1). With duplicates removed, 1978 articles were screened, 1836 excluded, and 142 retrieved in full text. As per Figure 1, 16 articles met the criteria for inclusion, and were retained in the final analysis. The remaining 16 articles included data from 18,352 children and adolescents aged 5–17 years [20,21,22,23,24,25,26,27,28,29,30,31,32,33,34,35], from 12 different countries, over four continents. Fifteen of the included articles utilised a quantitative descriptive research design, with one article adopting a mixed-methods approach [28]. Twelve articles presented data from cross-sectional studies [20,21,23,24,26,27,28,29,30,31,33,35], with a further four longitudinal studies capturing data within studies already commenced prior to the emergence of COVID-19 [22,25,32,34]. All articles included more than 100 participants, living under some degree of restrictions imposed to limit the spread of COVID-19. The majority of articles (15/16) presented data on correlates of PA, with only eight (50%) identifying correlates relating to sedentary time/behaviour. Individual study characteristics and overall MMAT scores for the included articles are provided in Supplementary Table S2. A full breakdown of the MMAT quality assessments with supporting justification is presented in Supplementary Tables S2 and S3.

**Figure 2:**
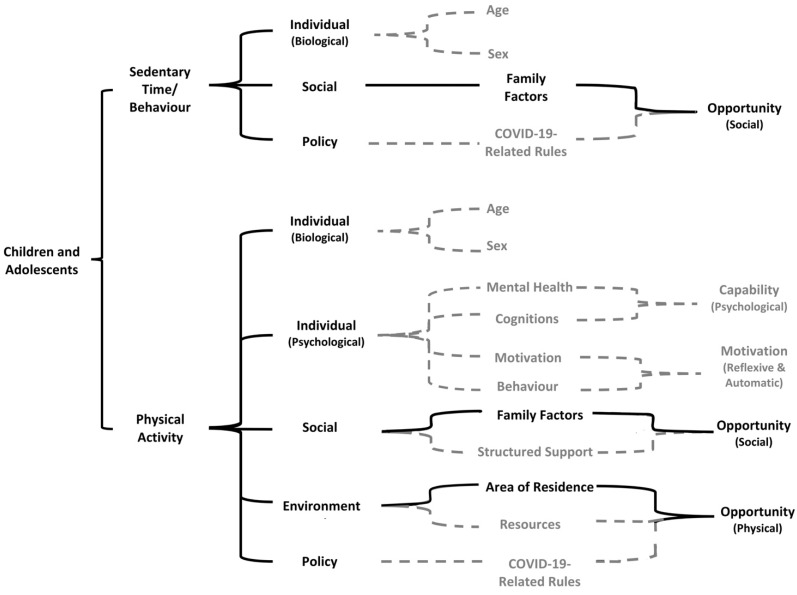
Socioeconomic model of correlates of PA and sedentary time/behaviour during the COVID-19 restrictions. Solid lines reflect the correlates and pathways for which the strongest evidence was identified, whilst those shown with dashed lines indicate those pathways and correlates with weaker evidence.

In line with the dimensions of Sallis et al.’s [10] socioecological framework, a narrative synthesis of the findings is presented, as well as mapping both PA and sedentary time/behaviour, where possible, into a socioecological model (Figure 2). More specifically, variables within individual, social, environmental and policy domains were mapped against the behaviour change mechanisms of the COM-B framework [11].

### 3.1. Individual—Biological Factors

#### Age and Sex

The significance of *age* and *sex* on the PA levels of children and adolescents during the pandemic is unclear. There is some evidence that increasing *age* was associated with lower PA levels during the pandemic [30,31,33], but this finding was not universally observed [25,35]. During the pandemic, younger children were more likely to participate in increased outdoor activities compared to adolescents [26] and engage in more unstructured PA, such as cycling, scootering, skateboarding or roller blading compared to older children (aged 9–13 years) [20]. In contrast, older children (aged 9–13 years) were more likely to participate in circuit training and conditioning exercises and were around five times more likely to engage with team sport training sessions via remote streaming than younger children (aged 5–8 years) [20]. Sex differences in PA during the pandemic were identified by several [20,22,28,32,35], but not all [25,26,30], studies, with those that did report a sex difference finding that the direction of the difference often varied depending on the specific type of PA undertaken. 

In terms of sedentary time/behaviour, older children were more likely to have higher levels of screen time [20,26,33], and overall sedentary or sitting time [30,33] when compared with younger children, with positive correlations reported between the total amount and *age*. However, others reported that longitudinally, whilst the overall proportion of children whose screen time was ≥ 2 h/day increased from 66% to 88% during the pandemic, there was no difference in screen time between primary and secondary school children [25]. However, Medrano et al. [25] found sex differences, with boys spending, on average, an hour longer on screen-based activities than girls. As well as differences in the amount of screen time [25,26], the specific type of screen time also differed between sexes; boys engaged more with computer or video games while girls connected more with social media, the internet and communication networks [20].

### 3.2. Individual—Psychological Factors

#### 3.2.1. Mental Health

Associations were identified between PA and components of *mental health* and well-being in children [35] and adolescents [21,28,35], including loneliness, depression, low mood state and overall mental health. Specifically, lower levels of loneliness and depression were observed in more physically active adolescents [21], whereas depression, confusion, anger, and fatigue were significantly higher in both children and adolescents with low or decreased PA, and vice-versa [35]. For overall mental health, decreased PA during lockdown was associated with lower mental health status in adolescents [28].

#### 3.2.2. Cognition, Motivation and Behaviour

In adolescents, parental concerns, cognitions regarding the potential health implications of COVID-19, and a general lack of motivation led to reductions in their PA during the pandemic [28]. Conversely, awareness of the health benefits of staying active, motivation gained from viewing PA as a source of entertainment, and positive prior PA behaviours, including having actively commuted to school, were associated with the adoption of more favourable PA behaviours during the pandemic [28].

### 3.3. Social Level Factors

#### 3.3.1. Family Factors

Multiple *family factors* related to the family dynamic or parental practices act as PA influencers. Specifically, co-engagement or encouragement from the whole family [21] or parents [27,28], having siblings (multi-child households) [26,30,33] or a dog [27], and parental marital status/co-habitation [27] were associated with increased PA or more outdoor play during the pandemic. Within-family conflict, for adolescents, decreased the likelihood of sufficient PA levels being achieved [22]. Parental capability to regulate their children’s behaviours (screen time, sleep patterns) [23] was associated with greater levels of PA, whereas parental levels of COVID-19 anxiety were, partially, related to decreased PA [24,28]. 

Studies exploring sociodemographic level factors focussed on parental socioeconomic status. A lower parental/household income was related to decreased outdoor PA both in children and adolescents relative to pre-COVID-19 [26], with a higher socioeconomic status correlated with increased outdoor PA [26,27,33] and greater access to suitable space [25]. The effects of parental age and education level remain debatable; it is suggested that having younger parents was associated with less decline in children’s PA and both indoor and outdoor play [27], and that a higher parental education level was positively related to children’s PA levels [22,25]. However, a lack of correlation between parental education and outdoor PA levels was also found [26]. 

Social factors were also revealed as the strong correlates of sedentary time/behaviour, specifically screen time. Living as part of a functional family unit [29] and/or having parents that were more interactive [29] or had a higher perceived capability to control screen time [23,29], negatively correlated with the amount of screen time. In contrast, parental practices deemed over-reactive or inconsistent in their approach to screen time limits [29] were positively correlated with screen time. Parental age (being > 43 years) was found to be related to less screen time [23], with lower household income predicting higher amounts [29]. Finally, higher parental COVID-19-related anxiety resulted in children being more likely to spend more time watching television, and two hours or more video gaming or using a computer [24].

#### 3.3.2. Structured Support

*Structured support* appears to be an important stimulant of PA, to a degree, across age groups. In adolescents, whilst a lack of guidance and access to organised PA (i.e., sport or exercise) limited opportunity and participation, seeking out positive role models acted as a facilitator [28]. Structured guidance, achieved primarily through technology-driven methods, also provided positive supportive approaches that were correlated to PA [20,27].

### 3.4. Environmental Factors

#### Area of Residence and Resources

The physical structure of a child or adolescents’ *area of residence* affected their PA engagement. Access to outdoor space was associated with increased levels of PA [20,23,25,27,33]. Living in a house [27], particularly compared to an apartment [26], and/or having access to large outside space [20,33] demonstrated a positive impact on PA. High dwelling-density and proximity to major roads was associated with reduced outdoor PA in children but not adolescents [26]. However, whilst the closure of roads and a reduction in traffic was observed as a way for adolescents to be able to go out into their neighbourhoods more, the closure of schools, recreation facilities, and cancellation of sports resulting in a loss of access to *resources* (facilities, equipment and coaching), was among the most cited barriers to PA in the majority of this population [28].

### 3.5. Policy Level Factors

#### COVID-19-Related Rules

The lack of normal routine associated with school was shown to be detrimental to adolescents. No physical education classes, after-school activities, or active travel due to the implications of *COVID-19-related rules*, coupled with perceptions of “too much extra schoolwork” and a lack of time, were related to reductions in PA [28]. This was not the case for all though, as some individuals found themselves with less to do and more available time [28]. Additionally, alterations to the work routines of parents were also related to PA and sedentary time/behaviour. Specifically, parents continuing to go to work outside of the home was associated with higher levels of PA in children [30,33] and less screen time in both children and adolescents [29].

## 4. Discussion

Through the exploration of socioecological correlates, this integrative review identifies the key mechanisms of behaviour change that may be conducive to challenging physical inactivity in children and adolescents, and stimulate re-engagement in PA, as we move forward and transition into the post-COVID-19-restriction era. Given the compelling evidence linked to the protective effect of childhood and adolescence PA on health in adulthood [36,37,38], advancing our understanding of the multi-level influences on PA, and where possible sedentary time/behaviour, is of paramount importance. Indeed, being able to effectively guide public health initiatives and policies, particularly for younger generations, could now be more important than ever. A recent systematic review concluded that most studies reported decreases in PA and increases in sedentary time/behaviours during the COVID-19 restrictions in both adults and children [7]. Furthermore, Runacres et al. [39] systematic review and meta-analysis demonstrated that children’s sedentary time increased the most (159.5 ± 142.6 min⋅day^−1^), compared to 126.9 ± 42.2 min⋅day^−1^ and 146.9 ± 22.0 min⋅day^−1^ in adults and older adults, respectively. Overall, children’s and adolescent’s PA was influenced by factors at each of the five levels: individual (mainly psychological), social (family and structured support), environmental (area of residence and resources), and policy. For sedentary time/behaviour, the findings suggest social level factors, namely family factors and particularly parental practices, may be primary influencers, followed by individual factors related to children’s mental health status and age, and policy factors, such as change in parent’s work circumstances. 

Contrary to pre-pandemic data [40], no age or sex associations with PA levels were evident. Whilst there was some indication that sedentary time/behaviour increased with age [20,29,30,31,33], the potential disparity between studies makes these findings difficult to interpret. Given that children are inherently sedentary during school hours [41], compliance with home-schooling regimes may, at least in part, explain the discrepancies reported in literature. It is, however, particularly noteworthy that age and sex differences were evident regarding the *type* of PA and sedentary time/behaviour [20] but it is unclear whether this was a direct result of the pandemic and its associated restrictions per se. Specifically, in accord with development preferences [42], younger children (aged 5-8 years) tended to spend time engaging in unstructured free play, whereas older children (aged 9–13 years) accrued the majority of PA via exercise, such as circuit training [20], likely a reflection of the more structured school-based approaches [43]. It is therefore unsurprising that access to outdoor space was of paramount importance for young children [20,26], with the loss of access to resources and facilities [28] being a key barrier for adolescents. The provision of exercise delivered through digital media may combat any potential negative consequences to adolescents’ overall PA levels, particularly those from disadvantaged backgrounds [44]. Regarding sex, boys were more likely to participate in sports practice and training and engage with computer games, whereas girls primarily participated in activity classes and lessons and used social media [20]. Given that self-perceptions of physical competence, motivation and enjoyment have been found to be longitudinal predictors of PA in young people [45], promoting preferred and previously experienced age- and sex-specific types of PA may enhance children and adolescents’ motivation and capability to be physically active. 

In adolescents, prior PA behaviours, including active commute to school, perceived health benefits of staying active and motivation from viewing PA as a source of entertainment were associated with adoption of more favourable PA practices during the pandemic [28]. Whilst the strength of this relationship may have been, in part, influenced by other factors from the socioecological model (e.g., parental cognitions and behaviours), these findings are in line with previous research demonstrating that stimulating positive attitudes and perceptions around potential health benefits of staying active can be a powerful driver for influencing intentions and PA behaviours in adolescents [46]. Future interventions should therefore seek to integrate such components to enhance long-term adherence. 

In accord with our findings in adults and older adults [14], clear associations were identified between components of mental health and PA both in children [35] and adolescents [21,28,35]. The relationship between mental health and PA is bi-directional, where poor mental health can be both a consequence or a cause of being less physically active [47]; poor mental health was reported as an important factor contributing to lower PA during lockdown [48]. The cross-sectional design of studies included in this review precludes specific analyses and interpretation; further longitudinal studies are required to clarify the specific mechanisms of action and the most pertinent intervention targets. Contrary to the majority of research reporting negative associations between sedentary time/behaviour and mental health in children and adolescents (for a review of reviews see Biddle and Asare [49]), no studies included in this review explored this relationship. 

Family dynamics and parental factors were of critical importance. These were observed to be determinants of both PA and sedentary time/behaviour on multiple levels, irrespective of age [21,23,27,28,29,30,33]. Similar to previous research [50,51], this review found that parental support, including co-participation and encouragement [21,27,28] and parental ability to regulate their children’s behaviours such as sleep and screen time [23], are key correlates of children and adolescents’ PA and sedentary time/behaviour behaviours. Among the strongest correlates was parental co-participation [27], suggesting that targeting co-participation, whilst respecting public health restrictions, may offer an opportunity to enhance children and adolescents’ PA behaviours during any ongoing and future restrictions in this or indeed future pandemics [52]. 

Associations between socioeconomic status-related factors and PA and/or sedentary time/behaviour were frequently identified, particularly access to outdoor space [20,22,26,27,33] and living in a house (as opposed to a flat) [27]. This is particularly concerning, suggesting that the COVID-19 pandemic has exacerbated pre-existing inequalities. Whilst it could be argued that lack of access to outdoor spaces, resources, and facilities would reverse as the restrictions ease, it is likely that these already disproportionately inactive communities may be further affected by poor health and a further decline in PA [44]. Although multi-level studies can inform future intervention strategies and policies through the identification of the key correlates of PA, it is pertinent to note that the impact of COVID-19 on the determinants of health, including PA, in disadvantaged communities is scant [44]. Further research is therefore required specifically focusing on identifying modifiable correlates of PA and sedentary time/behaviours. 

Finally, COVID-19-related restrictions associated with school closures (for the majority of young people), including cessation of physical education classes, after-school activities and active travel to school, had the expected negative impact on PA and sedentary time/behaviours in both children and adolescents [28]. However, interestingly, parental work patterns that remained relatively unchanged (going out to work) were associated with higher PA levels in children [30,33] and less screen time in both children and adolescents [29]. Although further interpretation of this finding is precluded given the lack of data, this further highlights the importance of parental control and practices on PA and sedentary time/behaviour of their children. 

### 4.1. The COM-B: Behavioural Targets and Policy Recommendations

As mapped in Figure 2, during the COVID-19 pandemic *opportunity* (social and physical) on a social and environmental level was the primary mechanism of change identified in children and adolescents. Whilst *capability* (psychological) and *motivation* (reflexive and automatic) are also highlighted within the PA section of the model, the level of evidence behind these themes limits their significance. This review identified associations between PA and components of mental health and well-being in children [35] and adolescents [21,28,35]. Although the small volume of evidence behind these themes potentially limits the strength of interpretation, overall, better mental health status was associated with being more physically active during the COVID-19 pandemic. Furthermore, higher PA levels was a predictor of less COVID-19 stress-related loneliness and depression in adolescents [21].

It is clear that removing the physical opportunity to be active during the initial period of pandemic control restrictions, in line with the findings observed in adults and older adults [14], also had detrimental consequences for children and adolescents. It is perhaps pertinent to note the potential difference between the relative duration of the pandemic-related restrictions and the long-term ramifications on PA and sedentary time/behaviour. If appropriate interventions and policies are not implemented to enhance, or at least re-instate pre-pandemic opportunities to be physically active, the effects on children and adolescents, particularly those at greatest risk (i.e., poor health-behaviour patterns; low socioeconomic status) may be amplified. In accord with an integrative review in adults and older adults [14], such oversights could widen socioeconomic health-related disparities, though the cross-sectional nature of the studies conducted to date, coupled with the reliance on subjective recall measures, makes this assertion difficult to quantify. For this population group, future policy also needs to consider other social factors, specifically, the potentially important role of strategies that promote co-participation, and the exploration of the provision of options, including through digital media platforms, that do not further disadvantage those from lower socioeconomic backgrounds.

### 4.2. Strengths and Limitations

This review was thorough, structured, and designed to encapsulate all relevant literature. However, there are some notable limitations. Only studies published in English were included, and only a small number of studies met the inclusion criteria; this is particularly pertinent to the data relating to sedentary time/behaviour that was subsequently available for analysis. Indeed, all results reported in the included studies were reliant on self-reported measures of PA and sedentary time/behaviour, which are known to be inherently susceptible to bias and under- or over-reporting when used by children [53]. Reliability of outcomes is further compounded by the participants needing, in some instances, to also recall pre-COVID-19 behaviour patterns [20,28], or the data being generated from parental recall [20,23,24,26,27,29,30,31,33]. It is, however, pertinent to note that whilst the use of device-based measures was limited, such online methods likely provided the most appropriate approach during the unprecedented, and indeed unpredictable, pandemic. Furthermore, the generalisability of the developed model is limited by the outcome variables utilised in the included studies, the limited number of longitudinal studies, and observational nature of the data. Therefore, whilst associations can be inferred, causation cannot.

## 5. Conclusions

The underpinning factors associated with the detrimental effects of the enforced COVID-19 restrictions on children and adolescents’ PA and sedentary time/behaviour across the world are complex and multi-faceted. Future behaviour-change interventions seeking to enhance young people’s PA and decrease sedentary time/behaviours during any periods of enforced restrictions should primarily limit restrictions to opportunities on a social and environmental level. *Capability* (psychological) and *motivation* (reflexive and automatic) may also be important for enhancing, or minimizing, the reduction of PA, though further evidence of these factors is warranted.

## Figures and Tables

**Table 1:** Study inclusion/exclusion criteria.

Variable	Inclusion Criteria	Exclusion Criteria
Population or participants and condition or interest	Children and adolescents aged 5–17 years Any sex/gender Not restricted to the UK	Studies including children aged <5 years (where separation of data was not possible) Studies including adults aged ≥ 18 years (where separation of data was not possible)
Intervention or exposures	Exposure to the COVID-19 pandemic, containment, and mitigation strategies	Studies that involve non-COVID-19 related pandemics, such as SARS or MERS
Comparison or control groups	No restrictions	
Outcomes of interest	Data/information, qualitative or quantitative, relating to correlates of PA and/or sedentary time/behaviour during the COVID-19 pandemic	No data relating to the pandemic phase or restrictions in place available Studies only including empirical data on volume of or changes in volume of PA or sedentary time/behaviour Data pooled from multiple different countries (where separation of data was not possible)
Setting	Any community setting	
Study designs	Any randomized, non-randomized, qualitative, or mixed methods study design providing original results	Studies not providing original results, such as systematic reviews, meta-analysis, general reviews, or editorials

COVID-19: novel coronavirus disease 2019; PA, physical activity; MERS, Middle East respiratory-system related coronavirus; SARS, severe acute respiratory syndrome; UK, United Kingdom.

## Data Availability

The data that support the findings of this study are available from the corresponding author upon reasonable request.

## Supplementary Materials

The following are available online at https://www.mdpi.com/article/10.3390/ijerph19031044/s1, Supplementary File S1: Example of search strategy for SPORTDiscus; Table S1: Study characteristics; Table S2: Coding criteria for the Mixed Methods Assessment Tool; Table S3: Quality assessment results from Mixed Methods Assessment Tool.
