# Supplementary material for: Moving Forward: Understanding Correlates of Physical Activity and Sedentary Behaviour during COVID-19 in Children and Adolescents—An Integrative Review and Socioecological Approach
Source: Int J Environ Res Public Health. 2022 Jan 18;19(3):1044. doi: 10.3390/ijerph19031044 (PMC8834078; doi:10.3390/ijerph19031044)
Supplement: Supplementary file 1 [file ijerph-19-01044-s001.zip › ijerph-1532418-supplementary/Supplementary Material/Supplementary File S1.pdf]

Supplementary File S1. Example of search strategy for SPORTDiscus

TI "coronavirus" OR AB "coronavirus" OR TI "COVID-19" OR AB "COVID-19" OR TI "COVID 19" OR AB "COVID 19" OR TI "SARSCoV2" OR AB "SARSCoV2" OR TI "SARS-CoV-2" OR AB "SARS-CoV-2" OR TI "SARS CoV 2' OR AB "SARS CoV 2" OR TI "novel coronavirus" OR AB "novel coronavirus" OR TI "COVID" OR AB "COVID" OR TI "n-CoV" OR AB "n-CoV"
AND
TI "physical activ*" OR AB "physical activ*" OR TI "exercise" OR AB "exercise" OR TI "physical function" OR AB "physical function" OR TI "physical performance" OR AB "physical performance" OR TI "sport" OR AB "sport" OR TI "recreation" OR AB "recreation" OR TI "active travel" OR AB "active travel" OR TI "sedentary time" OR AB "sedentary time" OR TI "sedentary lifestyl*" OR AB "sedentary lifestyl*" OR TI "sedentary behavi*" OR AB "sedentary behavi*" OR TI "physical inactiv*" OR AB "physical inactiv*" OR TI " prolonged sitting" OR AB "prolonged sitting"
