# Supplementary material for: Moving Forward: Understanding Correlates of Physical Activity and Sedentary Behaviour during COVID-19 in Children and Adolescents—An Integrative Review and Socioecological Approach
Source: Int J Environ Res Public Health. 2022 Jan 18;19(3):1044. doi: 10.3390/ijerph19031044 (PMC8834078; doi:10.3390/ijerph19031044)
Supplement: Supplementary file 1 [file ijerph-19-01044-s001.zip › ijerph-1532418-supplementary/Supplementary Material/Supplementary Table S1.pdf]

Supplementary Table S1. Study Characteristics.

Authors / Year	Country	Study Design	COVID-19 Restrictions	Inclusion/Exclusion Criteria	Participants	Pertinent participant demographics	Outcome	Outcome measurement tools	Overall MMAT grade
Dunton et al. (2020) [20]	USA (35 states and District of Columbia)	Cross-sectional via parental completed self-administered online survey (convenience sample)	Closure of Primary and Secondary schools closed and fitness centres. Partial closure of parks, trails, playgrounds, and beaches	Inclusion - 18+ years, speak and read English, USA resident, parent/ guardian of child (aged 5–13 years) with at least 50% custody and planned custody for next 12 months	*n* = 211,<br>Mean age children = 8.71 years (σ 2.58).<br>Mean age parents = 42.05 years (σ 5.34)	Parental sample stated to be mainly white, non-Hispanic female college graduates<br>82.46% of parents married<br>68.25% household income $95,000+	PA<br><br>Screen time and sitting time	Individually constructed questionnaire (validated)	****
Ellis, Dumas, et al. (2020) [21]	Canada	Cross-sectional via self-administered online survey	Varied between states, from stay-at-home orders to social distancing with variations on what facilities were closed.	Inclusion - High school students, 16-18 years for online recruitment. 14-18 years if already part of longitudinal study	*n* = 1054<br>Mean age = 16.68 years (σ 0.78)<br>Range 14-18 years	76.4% female<br>65.7% white<br>67.6% living with both parents	Time in daily activities<br><br>PA<br><br>Loneliness	Individually created scale (child completed)<br><br>Godin PA Questionnaire<br><br>Revised UCLA Loneliness Scale	****

Table S1. Study characteristics continued.

Authors / Year	Country	Study design	COVID-19 restrictions	Inclusion/exclusion criteria	Participants	Pertinent participant demographics	Outcome	Outcome measurement tools	Overall MMAT grade
Gilic et al. (2020) [22]	Bosnia and Herzegovina	Longitudinal via printed questionnaires and PA fitness level testing (baseline). Self-administered online survey (during pandemic)	Social distancing /facility closures/restricting public gatherings	Inclusion - Aged 15-18 years, attending high school	*n* = 688<br>Mean age = 17 years	322 females<br>65% lived in urban areas<br>Attended regular PE classes 2x/week pre-COVID-19	PA	Physical Activity Questionnaire for Adolescents (child completed)	*****
Guerrero et al. (2020) [23] (Linked to [26 and 27])	Canada	Cross-sectional via parental completed self-administered online survey	Varied between states, from stay-at-home orders to social distancing with variations on what facilities were closed.	Inclusion - Adult/parent of at least 1 child (5-11 years) or youth (12-17 years) in household.<br>Exclusion - Households where family member had contracted COVID-19 or family were in self-isolation	*n* = 1472<br>Mean age children = 8.1 years<br>(σ 2.0)<br>Mean age youth = 14.9 years<br>(σ 1.7)<br>Mean age parents = 45.12<br>(σ 7.55)	Parents<br>54% female<br>84.1% married/common law<br>70.1% employed full time<br>72.4% college/university degree.	PA<br><br>Screen time	Items from the Canadian Health Measures Survey	****

Table S1. Study characteristics continued

Authors / Year	Country	Study design	COVID-19 restrictions	Inclusion/exclusion criteria	Participants	Pertinent participant demographics	Outcome	Outcome measurement tools	Overall MMAT grade
McCormack et al. (2020) [24]	Canada (Calgary)	Cross-sectional via parental completed self-administered online survey	Stay-at-home order	Parent of at least 1 child (5-17 years).	*n* = 328<br>Mean age children 10.8 years (σ 4.0)<br>Mean age parents = 42.6 years (σ 8.8)	67.1% female<br>58.2% degree of higher<br>53% working full-time<br>56.4% Caucasian<br>82.6% married	Parental anxiety<br>Perceived change in child's PA and sedentary behaviour<br>PA<br>Screen time	Individually constructed questionnaire (validated)	***
Medrano et al. (2020) [25]	Spain	Longitudinal via self-administered online survey	Stay-at-home order	Inclusion - Children and Adolescents aged 8-16 years attending school participating in the MUGI project.<br>Exclusion - Pupils with an intellectual or physical disability preventing response to lifestyle questionnaire	*n* = 113<br>Mean age = 12.0 years (σ 2.6)	48.7% female<br>51% not meeting PA recommendations pre-COVID-19	PA<br>Screen time	The Youth Activity Profile<br>The Youth Activity Profile (child completed)	****

Table S1. Study characteristics continued.

Authors / Year	Country	Study design	COVID-19 restrictions	Inclusion/exclusion criteria	Participants	Pertinent participant demographics	Outcome	Outcome measurement tools	Overall MMAT grade
Mitra et al. (2020) [26] (Linked to [23 and 27])	Canada	Cross-sectional via parental completed self-administered online survey	Varied between states, from stay-at-home orders to social distancing with variations on what facilities were closed	Inclusion - Adult/parent of at least 1 child (5-11 years) or youth (12-17 years) in household. Exclusion - Households where family member had contracted COVID-19 or family were in self-isolation	*n* = 1472 Mean age children = 8.1 years (σ 2.0) Mean age Youth = 14.9 years (σ 1.7) Mean age parents = 45.12 years (σ 7.55)	89% college or university graduates 70% worked full-time 72% lived in houses versus apartments	Movement behaviours<br>Built environment	Individually constructed questions (validated)	****
Moore et al. (2020) [27] (Linked to [23 and 26])	Canada	Cross-sectional via parental completed self-administered online survey	Varied between states, from stay-at-home orders to social distancing with variations on what facilities were closed	Inclusion - Adult/parent of at least 1 child (5-11 years) or youth (12-17 years) in household. Exclusion - Households where family member had contracted COVID-19 or family were in self-isolation	*n* = 1472 Mean age children = 8.1 years (σ 2.0) Youth = 14.9 years (σ 1.7) Parents = 45.12 (σ 7.55)	54% female 84.1% married/common law 70.1% employed full time 88.7% college or university educated	Movement behaviours	Individually constructed questionnaire (validated)	****

Table S1. Study characteristics continued.

Authors / Year	Country	Study design	COVID-19 restrictions	Inclusion/exclusion criteria	Participants	Pertinent participant demographics	Outcome	Outcome measurement tools	Overall MMAT grade
Ng et al. (2020) [28]	Ireland	Mixed methods convergent cross-sectional via self-administered online survey	Stay-at-home order	Adolescents aged 12-18 years from 1 of 6 schools	*n* = 1214<br>Range 12-18 years	72% female<br>57.3% aged 12-15 years	PA	PA PACE+ instrument	***
							Change in PA	Individually constructed question (child completed)	
Ozturk and Yalçin (2021) [29]	Turkey	Cross-sectional via parental completed self-administered online survey	Stay-at-home-order	Parent of child aged 6-13 years	*n* = 1115<br>Mean age children = 9.03 years (σ 1.95)<br>Range 6-13 years	53.4% of children female<br>44% household over £400 per month/ 4000-6000 Turkish Lira	Screen Time	Individually constructed questionnaire (unclear validation)	****
							Parental Practices	Parental Practices Scale (parental completed)	
Pombo et al. (2020) - only data for 6–12-year-olds [30]	Portugal	Cross-sectional via parental completed self-administered online survey	Stay-at-home order	Parent/guardian of child < 13 years residing in Portugal	*n* = 932<br>Range 6-12 years	Unable to separate demographics for the specifically required age groups	PA	Individually constructed questionnaire (validated)	****

Table S1. Study characteristics continued

Authors / Year	Country	Study design	COVID-19 restrictions	Inclusion/exclusion criteria	Participants	Pertinent participant demographics	Outcome	Outcome measurement tools	Overall MMAT grade
Sá et al. (2020) only data for 6–12-year-olds [31] (Linked to [33])	Brazil	Cross-sectional study parental completed self-administered online survey	Social distancing and stay-at home-order but variable across five country regions	Parent of child aged < 13 years in the same household	*n* = 423<br>Range 6-12 years	Unable to separate demographics for the specifically required age groups	PA<br>Sedentary time and Screen time	Individually constructed questionnaire (validated)	***
Sekulic et al. (2020) [32] (Linked to [34])	Croatia (Split-Dalmatia)	Longitudinal study self-administered online survey	Social distancing/closure of schools and some public places/bans on public gatherings/no strict prohibition of different forms of individual training (e.g., running, cycling)	Healthy high school adolescent	*n* = 388<br>Mean age = 16.4 years (σ 1.9)<br>Range 15-18 years	126 female<br>35% involved in organised sports activities pre-COVID-19	PA (baseline March 20)<br>Baseline physical fitness (Sept 2019)	Physical Activity Questionnaire for Adolescents<br>Mandatory fitness panel	****

Table S1. Study characteristics continued

Authors / Year	Country	Study design	COVID-19 restrictions	Inclusion/exclusion criteria	Participants	Pertinent participant demographics	Outcome	Outcome measurement tools	Overall MMAT grade
Siegle et al. (2020) (only data for 6-12) [33] (Linked to [31])	Brazil	Cross-sectional study parental completed self-administered online survey	Social distancing and stay-at home-order but variable across five country regions	Parent/guardian of child < 13 years	*n* = 423	n = 285 6–9-year-olds<br>n = 138 10–12-year-olds<br>no other demographic data presented	Household Characteristics<br>PA<br>Domestic routines<br>Children routines	Individually constructed questionnaire (validated)	***
Zenic et al. (2020) [34] (Linked to 32]	Croatia (Split-Dalmatia)	Longitudinal study via self-administered online survey	Social distancing/closure of schools and some public places/bans on public gatherings/no strict prohibition of different forms of individual training (e.g., running, cycling)	High School Adolescents	*n* = 823<br>Mean age = 6.5 years (σ 2.1)	Minimal details; higher baseline PA in urban adolescents	PA (baseline March 2020)<br>Physical fitness (baseline Sept 2019)	Physical Activity Questionnaire for Adolescents<br>Mandatory fitness panel	****
Zhang et al. (2020) [35]	China	Cross-sectional study via self-administered online survey	Stay-at-home order	Grade 4 to 6 of primary schools	*n* = 9979<br>Mean age = 11.63 (σ 1.23)<br>Range 9-14 years	49% female<br>160 min PA/week in obligatory (PE) classes pre-COVID-19	PA<br>Mood	IPAQ – SF<br>Profile of mood states	***

[a] COVID-19 = novel coronavirus disease 2019; EQ-5D = EuroQol-5 dimension; GAD-7 = general anxiety disorder-7; HADS = hospital anxiety and depression scale; HRQoL = health-related quality of life; IPAQ = International Physical Activity Questionnaire; KSA, Kingdom of Saudi Arabia; QoL = quality of life; MET = metabolic equivalent; mins = minutes; MMAT = mixed methods assessment tool; MVPA = moderate-to-vigorous physical activity; n = number; PA = physical activity; PE = Physical Education; PROMIS = patient-reported outcomes measurement information system; RA =

rheumatoid arthritis; SB = sedentary behaviour; SD = standard deviation; SES = socioeconomic status; SF = short form; UK = United Kingdom; USA = United States of America; WHO = World Health Organization.

[b] Overall study quality was assessed using the Mixed Methods Assessment Tool (MMAT) and is reported using asterisks (*) as a descriptor, ranging from 1*, where 20% of the quality criteria have been met, to 5*, where 100% of the quality criteria have been met [14].
