# Supplementary material for: Moving Forward: Understanding Correlates of Physical Activity and Sedentary Behaviour during COVID-19 in Children and Adolescents—An Integrative Review and Socioecological Approach
Source: Int J Environ Res Public Health. 2022 Jan 18;19(3):1044. doi: 10.3390/ijerph19031044 (PMC8834078; doi:10.3390/ijerph19031044)
Supplement: Supplementary file 1 [file ijerph-19-01044-s001.zip › ijerph-1532418-supplementary/Supplementary Material/Supplementary Tables S2 and S3.pdf]

Table S2. Coding criteria for the Mixed Methods Assessment Tool

**Code Breakdown**	
Screening questions (for all types)	S1. Are there clear research questions?
	S2. Do the collected data allow to address the research questions?
1. Qualitative	1.1. Is the qualitative approach appropriate to answer the research question?
	1.2. Are the qualitative data collection methods adequate to address the research question?
	1.3. Are the findings adequately derived from the data?
	1.4. Is the interpretation of results sufficiently substantiated by data?
	1.5. Is there coherence between qualitative data sources, collection, analysis and interpretation?
2. Quantitative randomized controlled trials	2.1. Is randomization appropriately performed?
	2.2. Are the groups comparable at baseline?
	2.3. Are there complete outcome data?
	2.4. Are outcome assessors blinded to the intervention provided?
	2.5 Did the participants adhere to the assigned intervention?
3. Quantitative non-randomized	3.1. Are the participants representative of the target population?
	3.2. Are measurements appropriate regarding both the outcome and intervention (or exposure)?
	3.3. Are there complete outcome data?
	3.4. Are the confounders accounted for in the design and analysis?
	3.5. During the study period, is the intervention administered (or exposure occurred) as intended?
4. Quantitative descriptive	4.1. Is the sampling strategy relevant to address the research question?
	4.2. Is the sample representative of the target population?
	4.3. Are the measurements appropriate?
	4.4. Is the risk of nonresponse bias low?
	4.5. Is the statistical analysis appropriate to answer the research question?
5. Mixed methods	5.1. Is there an adequate rationale for using a mixed methods design to address the research question?
	5.2. Are the different components of the study effectively integrated to answer the research question?
	5.3. Are the outputs of the integration of qualitative and quantitative components adequately interpreted?
	5.4. Are divergences and inconsistencies between quantitative and qualitative results adequately addressed?
	5.5. Do the different components of the study adhere to the quality criteria of each tradition of the methods involved?

Table S3. Quality assessement results from Mixed Methods Assessment Tool

	1. Qualitative Studies					2. Randomized Controlled Trials					3. Non-Randomized Studies					4. Quantitative Descriptive Studies					5. Mixed Methods Studies						
	1.1.	1.2.	1.3.	1.4.	1.5.	2.1.	2.2.	2.3.	2.4.	2.5.	3.1.	3.2.	3.3.	3.4.	3.5.	4.1.	4.2.	4.3.	4.4.	4.5.	5.1.	5.2.	5.3.	5.4.	5.5.	Overall Score	Comments
Dunton et al. (2020) [20]																Y	Y	Y	U	Y						4	No other identifiable issues
Ellis, Dumas, et al. (2020) [21]																Y	Y	Y	U	Y						4	No other identifiable issues
Gilic et al. (2020) [22]																Y	Y	Y	Y	Y						5	4.4 Risk of non-response low as sample size known
Guerrero et al. (2020) [23]																Y	Y	Y	U	Y						4	No other identifiable issues
McCormack et al. (2020) [24]																Y	N	Y	N	Y						3	4.3 - measures deemed valid 4.4 high nonresponse rate
Medrano et al. (2020) [25]																Y	Y	U	Y	Y						4	Only 39% response rate, no details on what the PA measurement tools are.
Mitra et al. (2020) [26]																Y	Y	Y	U	Y						4	4.3 based on info from associated articles
Moore et al. (2020) [27]																Y	Y	Y	U	Y						4	No other identifiable issues. Qual analysis counted codes and displayed descriptively therefore qual MMAT not completed
Ng et al. (2020) [28]	Y	U	U	Y	Y											Y	Y	Y	U	Y	N	Y	Y	Y	U	3	5.1 There is no reference to the study being mixed methods 5.5 No info on who conducted the qualitative analysis, the position of the researcher within the data analysis etc 1.3 minimal information provided 1.1 process used justified

Ozturk & Yalçin (2020) [29]																Y	Y	U	Y	Y						4	Parent participation rates were 70.49%, 71.04% and 70.12%, respectively 4.3. No validation of measurement tool
Pombo et al. (2020) [30]																Y	Y	Y	U	Y						4	Survey pilot tested 4.2. ? mean bias towards younger participants
Sá et al. (2020) [31]																Y	N	Y	U	Y						3	4.2 sample is small relative to size of Brazilian population, 4.3 measures pilot tested
Sekulic et al. (2020) [32]																Y	Y	Y	U	Y						4	No other identifiable issues
Siegle et al. (2020) [33]																Y	N	Y	U	Y						3	4.2 sample is small relative to size of Brazilian population, 4.3 measures pilot tested
Zenic et al. (2020) [34]																Y	Y	Y	U	Y						4	No other identifiable issues
Zhang et al. (2020) [35]																Y	N	Y	U	Y						3	4.2 sample limited to children from one highly agricultural area, also states adolescents but only includes up to aged 14

Note: Y = yes; N = no; U = unclear
